# Green Spaces with Fewer People Improve Self-Reported Affective Experience and Mood

**DOI:** 10.3390/ijerph20021219

**Published:** 2023-01-10

**Authors:** Jordi Honey-Rosés, Oscar Zapata

**Affiliations:** 1 Institut de Ciència i Tecnologia Ambientals, Universitat Autònoma de Barcelona, Cerdanyola del Vallès, 08193 Barcelona, Spain; 2 Institute for Resources, Environment and Sustainability (IRES), University of British Columbia, Vancouver, BC V6T 1Z4, Canada; 3 School of Environment and Sustainability, University of Saskatchewan, Saskatoon, SK S7N 5C8, Canada

**Keywords:** affective response, field experiment, experiment, gender, green street, green space, mood, stress, wellbeing, public space

## Abstract

Calm and quiet green spaces provide health benefits for urban residents. Yet as cities become more densely populated, increasing public users to green spaces may reduce or moderate these benefits. We examine how increased pedestrian density in a green street changes self-reported wellbeing. We use a between subject experimental design that added public users as confederates in randomly selected periods over three weeks. We collect data on mood and affective response from pedestrians moving through the green street (*n* = 504), with and without our public user treatment in randomly selected periods. Mood and affective response are improved when experiencing the green street with fewer people. We find that an increased number of public users in the green space has a negative effect on mood, especially among women. We provide experimental evidence that self-reported wellbeing in urban green spaces depends on social context, and that there are gender inequities associated with changes in affective response. Although we only measure immediate impacts, our results imply that the health benefits of green spaces may be limited by the total number of users. This research contributes additional evidence that greener cities are also healthier cities, but that the benefits may not be equally shared between women and men and will depend on the social context of use.

## 1. Introduction

Green spaces provide urban residents with multiple health benefits [1,2] and there is evidence that a green city is also a healthy city [3,4,5]. The COVID-19 pandemic renewed interest in the healthy city and reappraised the value of urban green spaces [6,7]. While green spaces have long been a cherished place for urban refuge [8], the pandemic forced policy makers to scrutinize the relationship between urban density, green spaces, and health [8,9,10].

This renewed attention on the healthy city is also pushing scholars to understand the mechanisms and pathways by which green spaces may improve the health and wellbeing of urban residents [2,11,12]. The attention restoration theory posits that nature allows for recovery from attention fatigue [13]. Others emphasize improved environmental conditions, such as reduced noise, temperature, and air pollution or the absence of other environmental stressors [14,15,16]. Green spaces also provide urban residents with opportunities for physical activity [17] and socialization [18,19].

Regardless of the causal pathway studied, the literature on the health benefits of green spaces is largely based on observational studies with large datasets that have allowed researchers to identify strong associations between green space availability and health outcomes [11,20,21]. We have also learned from longitudinal studies that follow the health outcomes of large cohorts over time [22,23]. Researchers have tracked participants in their everyday lives to learn how self-reported measures of wellbeing, combined with salivary cortisol measurements, might correlate with exposure to green spaces [24,25].

Scholars have also used framed experiments to understand the relationship between nature exposure and health [26,27,28,29]. Early experimental work showed that walking in nature provided greater stress relief than walks in urban settings [13]. In some instances, researchers have found that exposure to forested, green, or natural environments provide measurable improvements in biomarkers and biophysical indicators of stress [29]. Even short-term visits to nature are found to relieve stress more effectively than the comparable exposure to built environments [30]. Researchers have even employed lab experiments to identify dose-response relationships between nature exposure and health outcomes [31].

This combination of observational and experimental work has slowly built a body of evidence on the health benefits of green spaces [32]. A systematic review of reviews shows particularly strong evidence that contact with natural environments improves emotional wellbeing and affect [4]. The emerging consensus on the likely health benefits from exposure to nature has led scholars to call for the inclusion of nature in public health planning [33].

As the benefits of creating new green spaces in cities are increasingly clear, urban planners are under pressure to provide more green spaces to more people [34,35]. Simultaneously, city leaders also feel pressure to increase urban residential density in order to make housing more affordable and meet sustainability objectives [36,37,38]. Yet paradoxically, increasing urban density is likely to add additional pressures to a city’s existing green infrastructure [35,39]. How might the health benefits associated with green spaces change as pedestrian density increases? Might the health benefits generated by green spaces be contingent on how many other users are present?

Of the various mechanisms and pathways by which green spaces may produce health benefits, the pathway on socialization has received less attention. Until now, scholars have emphasized that socialization is a key pathway for improved wellness in urban green spaces [19]. Green spaces provide residents with a place to greet neighbors, feel part of their community, mitigate loneliness, and reinforce their sense of belonging [19]. On the other hand, more users in a green space may also reduce the associated benefits related to fatigue recovery, mood improvement, or stress reduction [29]. These countervailing forces need quantification to more precisely estimate the health benefits of green spaces in dense urban environments.

Scholars from the recreation sciences have led efforts to understand the impacts of crowding on the user experience in parks, protected areas, and hiking trails [40,41,42,43]. A subset of this work has examined the user experience in urban green areas [44,45,46,47], largely finding that low use-levels are preferred over high use-levels [44,48]. In urban and green spaces alike, high human density can cause stress because of stimulus overload [49]. Furthermore, visitor preferences for stress relief in urban parks closely matches preferences for green areas more generally [48].

This literature relies mostly on choice experiments with digitally calibrated images that allow users to choose among recreation experiences to estimate preferences and trade-offs [42,44,48,50,51]. While this body of work has advanced our understanding of user preferences in green spaces, it samples from a subset of users that self-select themselves into the green destination. Stated preferences methods also produce hypothetical rather than actual choices. Lastly, the research subjects in choice experiments are aware that their choices are being studied. To our knowledge, no research has examined the health impact of public use-levels using a between subject experimental design in a field setting. In this study we record changes to mood and affective response produced by increased pedestrian density in a green street.

The aim of this study was to measure the changes in mood and affective response produced by increased pedestrian density in a residential green street [52,53,54,55,56]. While previous work has used other methods to measure the health benefits of exposure to nature, we look at this question using a field experiment in a real-world setting in which research subjects are unaware that they form part of an experiment. Our research design allows us to record the immediate health impacts of increasing pedestrian density in a public green space.

## 2. Materials & Methods

### 2.1. Study Area

**Figure 1:**
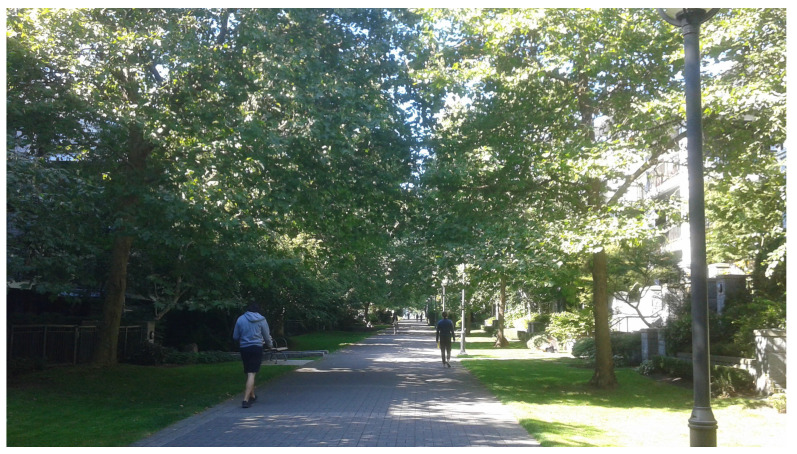
The studied green street in Vancouver, Canada.

We developed a field experiment in a real world setting to study the impact of increased pedestrian density in a green street. We conducted our experiment in a pedestrianized green street that traverses a residential community on the southern edge of a university campus in Vancouver, Canada (Figure 1). The study area has rectangular dimensions: 155 m long and 20 m wide. The green street is closed to vehicular traffic and straddled on both sides by multi-unit residential buildings, resulting in a quiet and calm pedestrian promenade. The green street is abundant in vegetation and grass, making it an inviting place for rest, contemplation, and relaxation. Twelve native maple trees (*Acer macrophyllum*) provide canopy cover and shade in the warm summer months. The sound of water from a fountain contributes to a peaceful environment. Pedestrians may find three public benches that fit three people each and there is additional seating on a fountain ledge that may seat eight more individuals. This area offers a space for mixing between residents and the academic community.

We observed the space prior to our experiment to collect exploratory data on current conditions and use. We observed an average of 72 users per hour, with most users moving through the site rather than staying. Approximately 90% of users were pedestrians, of which 52% walked alone, 35% in pairs, and 12% in groups of three or more. The site is quiet and calm, with long periods in which no one may be present. We noted more female than male users. Approximately one quarter of the users are residents, as evidenced by entry and exit patterns to the residential units adjacent to the green street. These exploratory observations were not used in the formal analysis, but rather collected to understand general patterns in use prior to developing the experiment.

### 2.2. Experimental Design

We designed a between subject field experiment to record the change in mood and affective response of pedestrians using a green street when increasing the total number of users in the public space. In particular, we aimed to learn if there might be changes to self-reported affective response, especially mood and stress. We used a time-randomization procedure to determine which subjects would be assigned to the treatment or control condition. We used a block randomization to ensure that the hours assigned to treatment and control conditions were balanced by the day of the week and time of day. During the control conditions, no additional public users were added to the green street. In the hours randomly selected as the treatment condition, we added between 10 and 16 individuals to the green street. For this purpose, we recruited students to be confederate public users who rotated between three activities: (1) walking, (2) quiet staying activity (reading, quiet enjoyment, or using electronics), and (3) social staying activity (i.e., chatting, playing card games). Three groups of confederates rotated between each activity, ensuring that all activities were taking place simultaneously. They followed strict protocols and guidelines to avoid bringing attention to themselves or revealing that they were part of an experiment. The age, gender, and racial composition of the confederates reflected what is found in the university community as they were predominately female (61%), White (45%), and Asian (42%).

We collected survey responses from pedestrians walking through the green street from 10:00 to 16:00 h from Monday to Friday during a three-week period in August 2018. We obtained written consent from survey respondents to participate in the research. Half of the 90 h of data collection were randomly devoted to the treatment conditions and half to the control condition. We collected word descriptors by asking respondents to share two words that described the place. Collecting word descriptors aimed to capture how the addition of public users might alter perceptions of the green street. The word descriptors were grouped by core meaning and analyzed with the Poisson regression and Chi-squared tests.

For each survey respondent, we collected the age, gender, university affiliation and the frequency in which they visited the green street (often, occasionally, rarely, never). We hypothesized that frequent visitors, (i.e., neighbours), would be most sensitive to the experimental treatment.

Finally, we recorded the affective response with Likert scored statements: today I am in a good mood (mood); today I feel a bit stressed (stress); today this is an ideal place to relax (relax); today this place makes me feel peaceful (peaceful); each statement was scored from 1 to 7. We compared scores for treatment and control responses using an ordered Logit model with clustered standard errors. As a between subject design, we are interested in comparing the responses of those who responded to the survey in the pedestrian treatment condition to those who responded in the control condition. Therefore, we are particularly interested in the coefficient on treatment in the ordered logit model that predicts the affective response obtained in the surveys. We employ STATA as our statistical software to perform the analyses. Our pre-analysis plan was deposited in the study registry managed by Evidence in Governance and Politics (20180810AA) before data collection, and we received approval from the Behavioural Research Ethics Board of the University of British Columbia (H18-01446).

## 3. Results

We obtained 506 survey responses during the three-week period, of which 252 surveys were in the control condition and 254 surveys in the treatment condition (Supplementary Material Table S1. We observe comparable groups with similar characteristics by age, gender, university affiliation, and frequency of visit (Supplementary Material Table S2).

We find experimental evidence that self-reported mood and affective response decreases with more people present in the green street. The impact of the pedestrian treatment on self-reported wellbeing is particularly strong among women. Mood and affective response are higher when users enjoy the green street in relative solitude.

Increasing users in the public realm made pedestrians more likely to describe the space as *green*, *welcoming*, and *shaded*, but less likely to describe the place as *peaceful*, *open*, or *inspirational*. More generally, when public users are added, we observe a shift in word descriptors from *peaceful* to *green*, and this is consistent for both genders. In other words, with more people, the place became less *peaceful*, and the *green* condition became more salient. While this shift makes intuitive sense, the differences in word descriptors between the treatment and control groups are not statistically significant in a Chi-Squared test or Poisson regression.

Our core results concern the changes in self-reported wellbeing with the increased number of public users in the green street. To identify these changes, we examine the coefficients on treatment in our ordered logit model. We find significant and consistent effects on mood, in which a higher pedestrian density reduces mood in all models (Supplementary Material Table S3). Those surveyed in the green street without the additional public users report being in a better mood. These results are significant in aggregate but are particularly strong among women (Table 1). The effect on women is highly significant (*p* < 0.001), while the coefficient on *mood* for men is similarly negative but not significant. As a result, the total pedestrian effect on *mood* is driven by a large effect among women. At the same time, the negative effect on *mood* is consistent across users, regardless if they visited the site often, occasionally, rarely, or never.

More public users also increase levels of self-reported stress; however, these effects are only statistically significant among users who visit the site often, such as neighbours. This increase in stress among frequent visitors is consistent with our original hypothesis, since we expect neighbours to be more uneasy about increased public users in their neighbourhood.

We also find that more public users in the green street make people less likely to describe the green street as a place to relax. These results are consistent across gender and frequency of visit. As with mood, we find important gender differences in which women are again more sensitive than men to the increased number of public users. Finally, and as expected, pedestrians are less likely to feel *peaceful* under treatment conditions, however these results are not significant.

## 4. Discussion

We show that increasing the number of people using an urban green space will impact mood and affective response. In general, it appears that green spaces may have a calming effect on individuals in the absence of high levels of public use. In particular, increasing public users in green spaces dampens mood, especially among women. We provide novel experimental evidence on the impact of public use on the affective experience in urban green spaces. We build off of past work that has largely relied on other research methods [44,45,46].

While the experimental design allows us to generate unbiased estimates on the effect of increasing public users in a green space, our study has several limitations. We record only immediate changes to mood and affective response rather than any long-term effect. It is also unclear how our results might vary at night or in other spaces, cities, or cultures. Our results are most transferable to urban green streets, especially in residential settings that are perceived to be safe. Urban green spaces that are vastly different than our study site or are perceived to be unsafe might produce different findings.

A follow up study could improve on our research design by using a validated tool to measure mood and stress. Our use of simple and unambiguous agree/disagree statements provides a fast way of capturing mood and stress, but more sophisticated measures could also be used. Examples of sophisticated or objective measures of stress include blood markers, heart rate, oxygen consumption [57], cortisol levels [30,57,58], and arterial pressure [58]. Other studies use mental workload tasks to generate fatigue, and stress and identify their effects on performance [59,60]. Interestingly, the evidence of how subjective and objective measures of mood and stress compare is still inconclusive [57,61,62,63].

Our results are relevant for scholars who aim to understand the causal mechanisms and pathways by which green spaces provide health benefits [2,3,18,21,64]. In particular, our results speak to those who have put forward social life as a critical pathway for improved health benefits associated with green spaces [18,64,65]. While social interaction may benefit many green space users, we illustrate the trade-offs associated with increasing public users to green spaces. For many, the absence of people may assist in generating health benefits, including improvements in mood, affect, and stress recovery. Our study only narrowly measures the impact of adding public users on mood and affective response, without accounting for the other potential benefits produced if users knew one another or socialized. Therefore, it is unclear how the benefits associated with socialization may compensate for negative impacts found here. More assumptions and a different research design would be needed to quantify all of the trade-offs and changes in affective response associated with increasing more users in green spaces.

While researchers have made progress on assessing the quality of green spaces [65], and others have emphasized the need to pick apart the dimensions of green spaces that are connected to mental health [66], the pandemic invites us to reconsider the total use or carrying capacity of green spaces as a moderator that may impact mood, stress, and other measures of wellbeing. We see a need to incorporate total public use in models that aim to estimate the health benefits of green spaces. We speculate that green spaces might have an implicit carrying capacity of users [67], or a critical threshold, above which health benefits are produced and below which the generated health benefits might be much smaller.

At the same time, our results are consistent with observational studies and framed field experiments that show a strong association between access to green spaces and health. We build on this literature by providing experimental evidence of how self-reported mood and other wellbeing indicators might change in green spaces with high levels of public use. Our results on mood are consistent with a systematic review of the public health evidence of exposure to natural environments, which found the strongest evidence for the reduction of negative emotions [4].

The results from our experiment also underscore the importance of the social context of green spaces. Our findings reinforce the idea that green spaces are socially embedded in cities. As a result, the health benefits produced are not merely a function of their urban design or landscaping, but also conditional on social context and relations [68], which may have heterogeneous spatial distributions [69]. Therefore, research studying the impact of green spaces on wellbeing should account for the number of users in these spaces and their relationships to each other. Planners would be well served by promoting a diversity of green spaces to distribute users and prevent overcrowding.

## 5. Conclusions

We aimed to learn how the social context of an urban green space might impact self-reported levels of stress and mood. We developed a novel field experiment using a between subject design in a real-world setting in which research subjects are unaware that they form part of an experiment. We find inequities by gender, in which women are more sensitive than men to the increased pedestrian density in green spaces. This research contributes additional evidence to the idea that greener cities are also healthier cities, but that the benefits may not be equally shared between women and men and will depend on the social context of use.

## Figures and Tables

**Table 1:** Changes in self-reported affective experience produced by the increased number of public users in a green street, by gender and frequency of visit.

		Gender		Frequency of Visits			
Outcome	All	Female	Male	Often	Occasionally	Rarely	Never
Variables	(1)	(2)	(3)	(4)	(5)	(6)	(7)
Mood	−0.493 **	−0.867 ***	−0.192	−0.529 *	−0.447	−2.053 *	−0.596
	(0.190)	(0.212)	(0.378)	(0.234)	(0.566)	(1.036)	(0.468)
Stress	0.255	0.080	0.556	0.717 **	−1.102	−0.117	0.001
	(0.184)	(0.237)	(0.431)	(0.208)	(0.646)	(1.012)	0.000
Relax	−0.420 ***	−0.521 **	−0.293	−0.211	−0.750	−0.853	−0.600
	(0.094)	(0.184)	(0.172)	(0.308)	(0.647)	(1.202)	(1.290)
Peaceful	−0.059	−0.340	0.156	−0.301	0.607	−0.627	−0.271
	(0.131)	(0.311)	(0.230)	(0.425)	(0.392)	(1.163)	(0.634)
Observations	506	244	262	224	104	61	114

Legend: * *p* < 0.05; ** *p* < 0.01; *** *p* < 0.001. Standard errors in parentheses.

## Data Availability

The datasets generated during and/or analysed during the current study are available from the corresponding author on reasonable request.

## Supplementary Materials

The following supporting information can be downloaded at: https://www.mdpi.com/article/10.3390/ijerph20021219/s1, Table S1: Mean results in treatment and control conditions (SD); Table S2: Covariate balance of full sample; Table S3: Robustness checks of ordered logit models.
