# Supplementary material for: Green Spaces with Fewer People Improve Self-Reported Affective Experience and Mood
Source: Int J Environ Res Public Health. 2023 Jan 10;20(2):1219. doi: 10.3390/ijerph20021219 (PMC9858984; doi:10.3390/ijerph20021219)
Supplement: Supplementary file 1 [file ijerph-20-01219-s001.zip › ijerph-2128950-supplementary.pdf]

Supplementary Material Table S1. Mean results in treatment and control conditions (SD).

	Control Condition	Treatment Condition
Today I feel a bit stressed.	2.794<br>(1.61)	3.099<br>(1.78)
Today I am in a good mood.	5.825<br>(1.12)	5.610<br>(1.15)
Today, this place is ideal to relax.	6.299<br>(1.02)	6.098<br>(1.22)
Today, this place makes me feel peaceful.	6.151<br>(1.15)	6.079<br>(1.08)

Supplementary Material Table S2. Covariate balance of full sample (n = 506). The similar characteristics in the treatment and control groups suggest that randomization was successful in creating two comparable groups.

Characteristics	Control (%) *	Treatment (%) *	Difference	*p*-value
Female	50.8	45.7	0.051	0.252
University affiliate	67.1	65.6	0.014	0.732
Age (years)	36.4	35.9	0.498	0.576
Frequency of visit	0.437	0.445	−0.009	0.842

Supplementary Material Table S3. Robustness checks of order logit models.

Outcome Variables	Robust SE (1)	Clustered SE (2)	Robust SE (3)	Clustered SE (4)
Mood	−0.387 *	−0.387 *	−0.493 *	−0.493 **
	(0.185)	(0.192)	(0.192)	(0.190)
Stress	0.125	0.125	0.255	0.255
	(0.188)	(0.193)	(0.196)	(0.184)
Relax	−0.360	−0.360 ***	−0.420 *	−0.420 ***
	(0.205)	(0.091)	(0.208)	(0.094)
Peaceful	−0.070	−0.070	−0.059	−0.059
	(0.185)	(0.124)	(0.198)	(0.131)
Controls				
Block variable	Yes	Yes	Yes	Yes
Female	No	No	Yes	Yes
Age group	No	No	Yes	Yes
UBC affiliation	No	No	Yes	Yes
Frequency of visit	No	No	Yes	Yes
Observations	506	506	506	506

Note: * $p < 0.05$; ** $p < 0.01$; *** $p < 0.001$. Standard errors in parentheses.
